# Supplementary material for: MiR-125b-5p Is Involved in Sorafenib Resistance through Ataxin-1-Mediated Epithelial-Mesenchymal Transition in Hepatocellular Carcinoma
Source: Cancers (Basel). 2021 Sep 30;13(19):4917. doi: 10.3390/cancers13194917 (PMC8508441; doi:10.3390/cancers13194917)
Supplement: Supplementary file 1 [file cancers-13-04917-s001.zip › cancers-1393031-supplementary.pdf]

cancers MDPI

Supplementary Materials: MiR-125b-5p Is Involved in Sorafenib Resistance through Ataxin-1-Mediated Epithelial-Mesenchymal Transition in Hepatocellular Carcinoma

Akihiro Hirao [1], Yasushi Sato [2,*], Hironori Tanaka [1], Tetsu Tomonari [1], Kensei Nishida [3], Misato Hirata [1], Masahiro Bando [1], Yoshifumi Kida [1], Takahiro Tanaka [1], Tomoyuki Kawaguchi [1], Hironori Wada [1], Tatsuya Taniguchi [1], Koichi Okamoto [1], Hiroshi Miyamoto [1], Naoki Muguruma [1], Toshihito Tanahashi [1], Tetsuji Takayama [1,*]

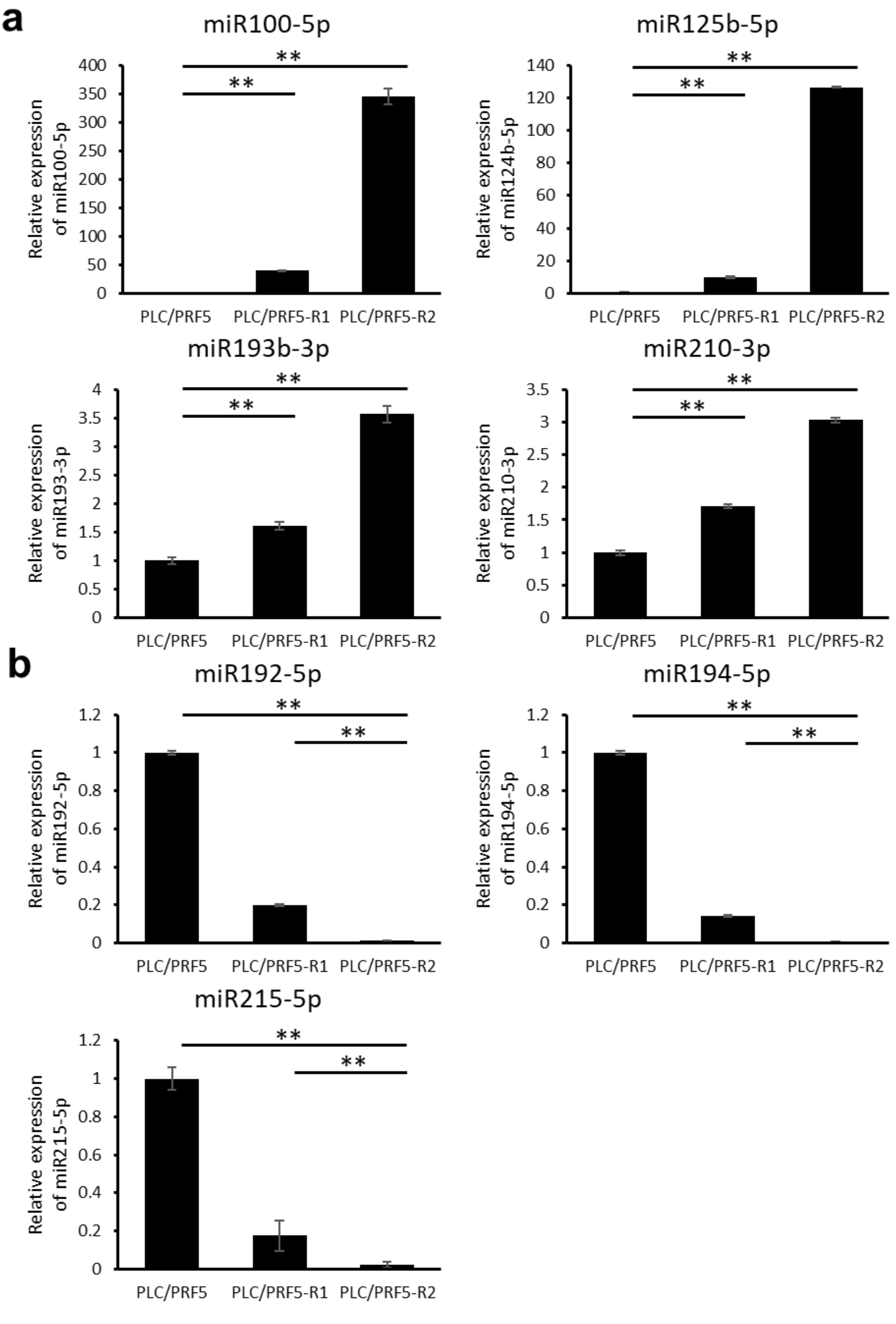

Figure S1. Validation of miRNA expression levels predicted by microRNA array in PLC/PRF5, PLC/PRF5-R1, and PLC/PRF5-R2 cells. The miRNA expression levels were determined by RT-PCR. (**a**) Upregulated miRNAs, (**b**) Downregulated miRNAs. Data are expressed as mean ± SD. **p<0.01.

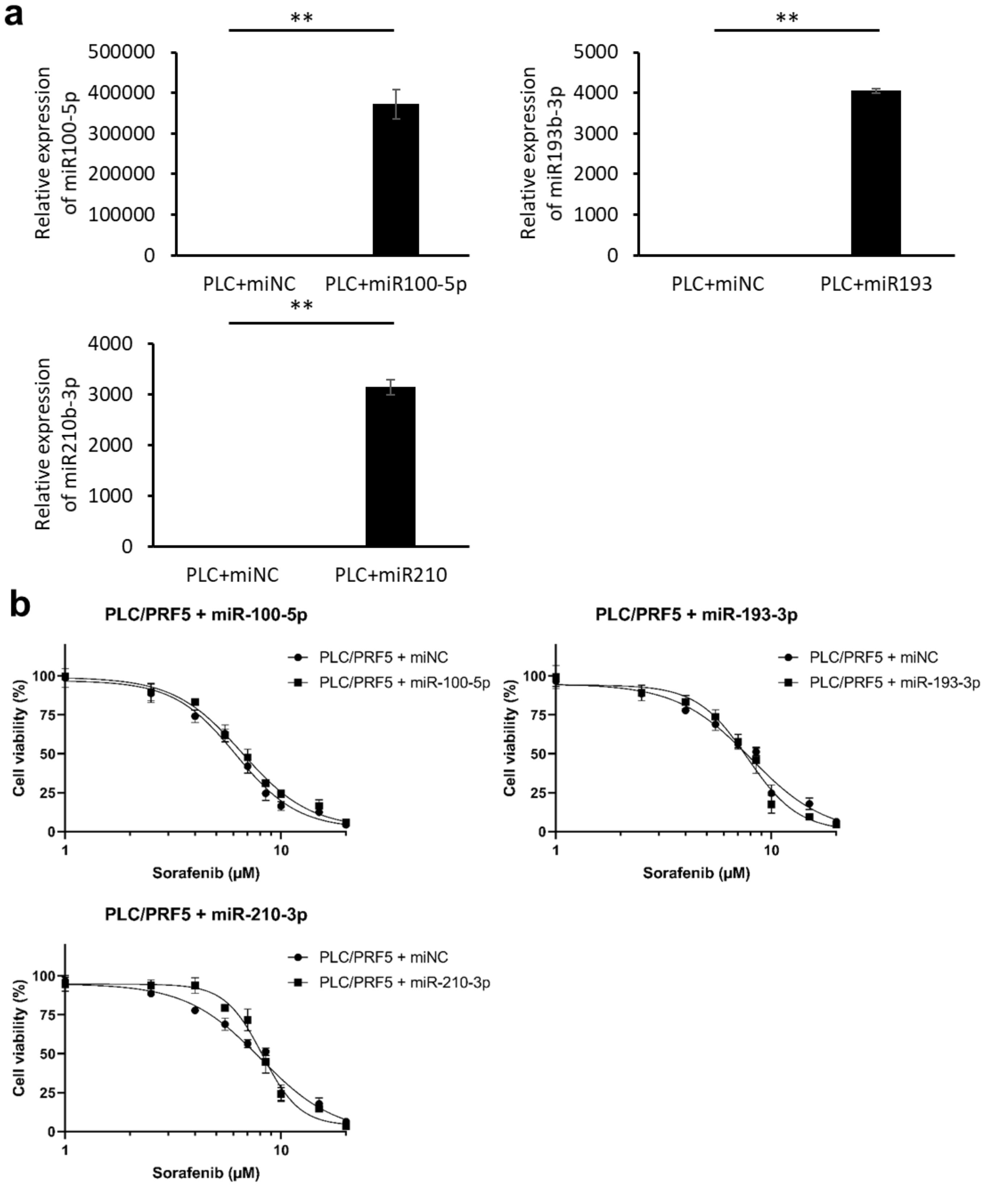

Figure S2. None of the miRNAs (miR-100-5p, miR-193, miR-210) had any effect on sorafenib resistance. (**a**) PLC/PRF5 cells were transfected with each miRNA (miR-100-5p, miR-193, miR-210) mimic or miNC, and the relative expression of miRNAs was measured by RT-PCR. **p<0.01. (**b**) The cells were treated with various concentrations of sorafenib, and cell viability was measured by WST assay. IC50 values against sorafenib were determined by non-linear regression analysis.

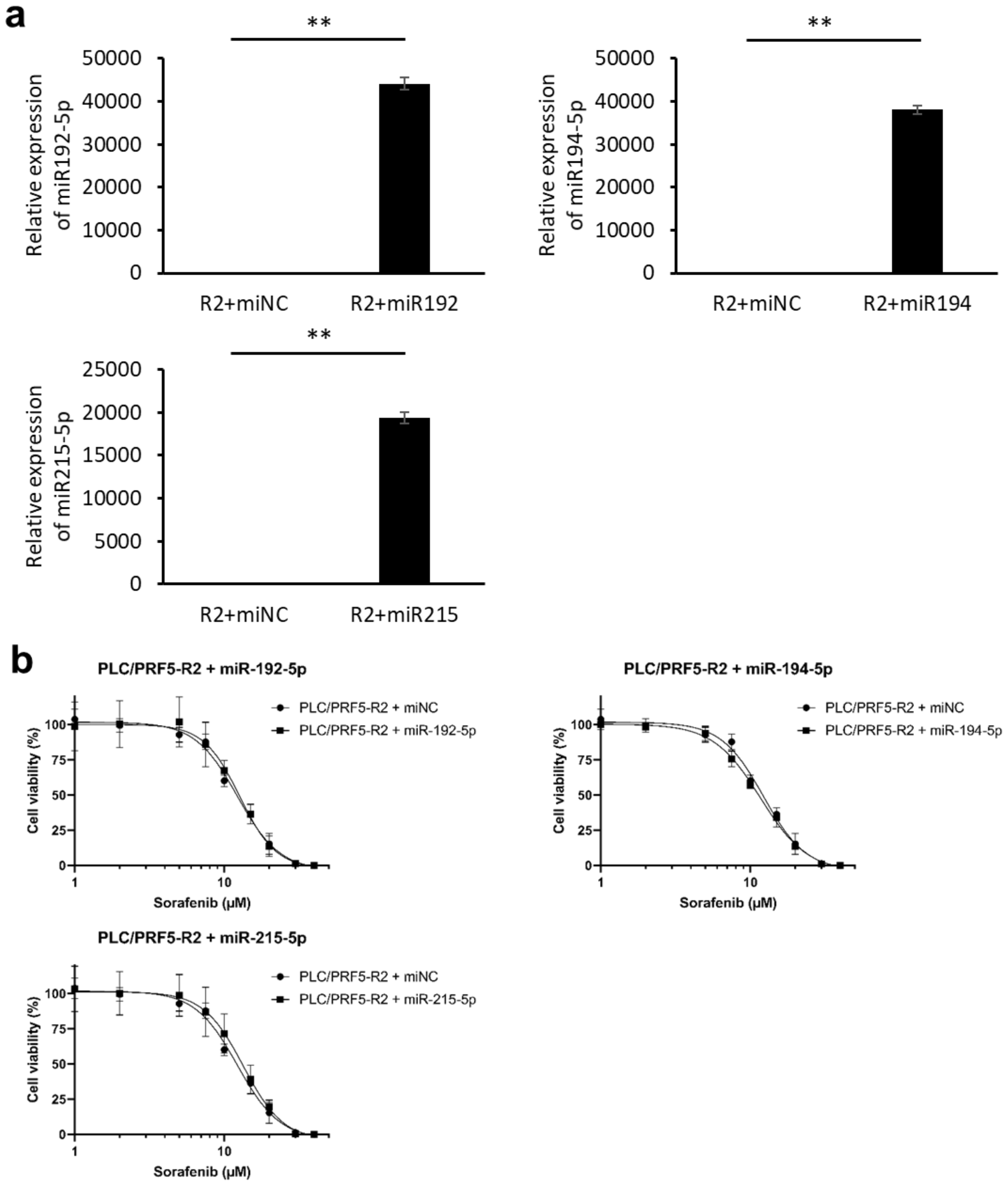

Figure S3. A None of the miRNAs (miR-192, miR-194, miR-215) had any effect on sorafenib resistance. (**a**) PLC/PRF5-R2 cells were transfected with each miRNAs (miR-192, miR-194, miR-215) mimics or miNC, and relative expressions of miRNA were measured by RT-PCR. **p<0.01. (**b**) The cells were treated with various concentrations of sorafenib, and cell viability was measured by WST assay. IC50 values against sorafenib were determined by non-linear regression analysis.

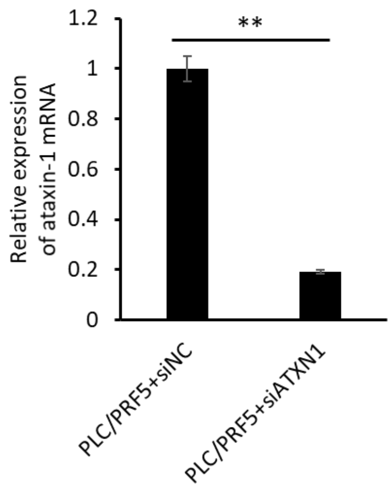

Figure S4. The relative mRNA expression levels of ataxin-1 in PLC/PRF5 cells transected with ataxin-1 siRNA (PLC/PRF5-siATXN1) and control siRNA. The ataxin-1 mRNA levels in PLC/PRF5-siATXN1 cells and PLC/PRL5 cells transfected with siRNA negative control were determined by RT-PCR. The values were compared and analyzed by Student's t test. **p<0.01.

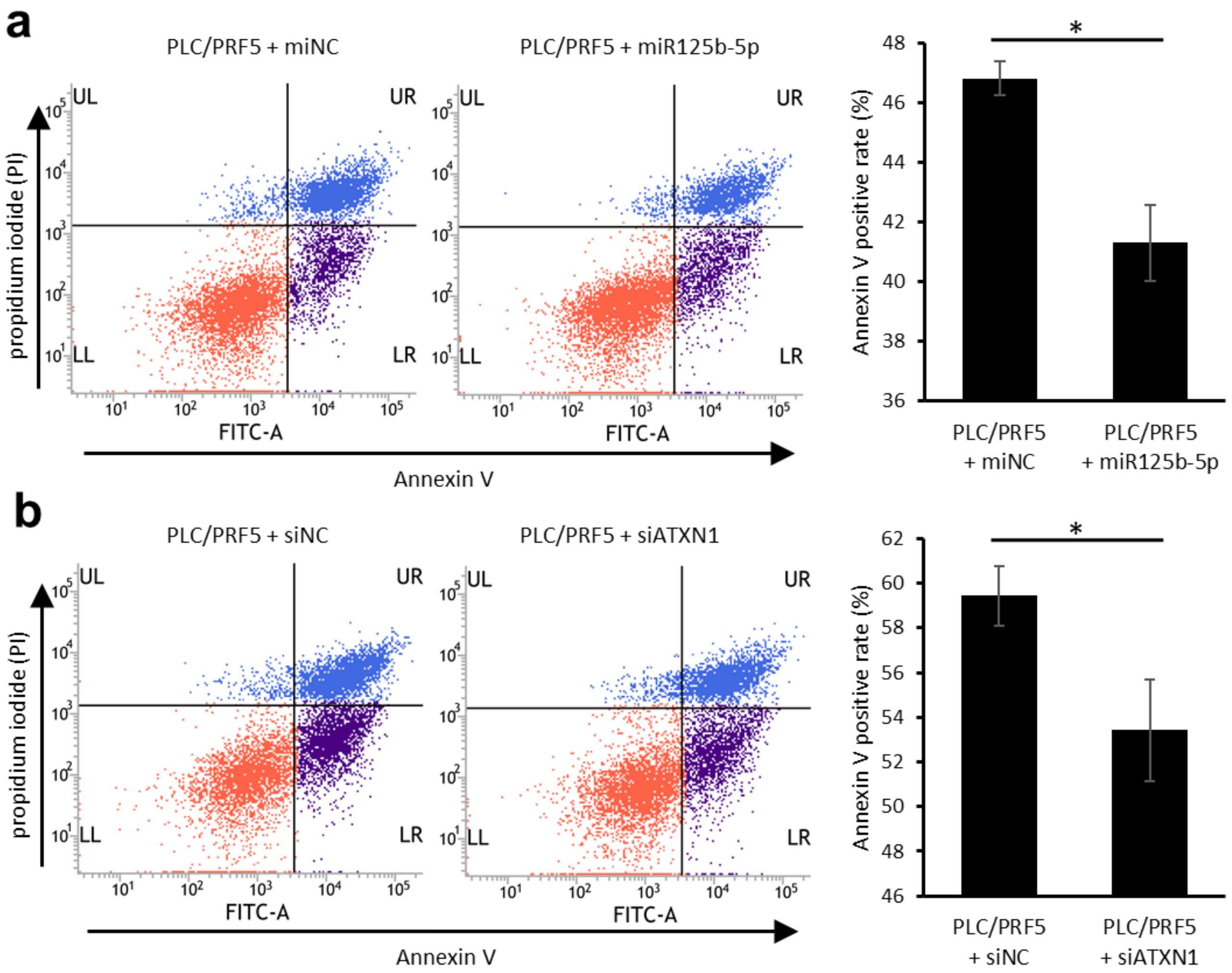

Figure S5. Apoptosis was suppressed in HCC cells after treatment with miR-125b-5p mimic or siRNA-ATXN1. (a) Flow cytometric analysis of PLC/PRF5 cells treated with miNC or miR125b-5p mimic for annexin V-FITC and propidium iodide (PI) staining of cells treated with sorafenib (10 μM). (b) Flow cytometric analysis of PLC/PRF5 cells treated with siNC or siATXN1. Data represent mean ± SD of triplicate experiments. *p <0.05 by Student's t test.

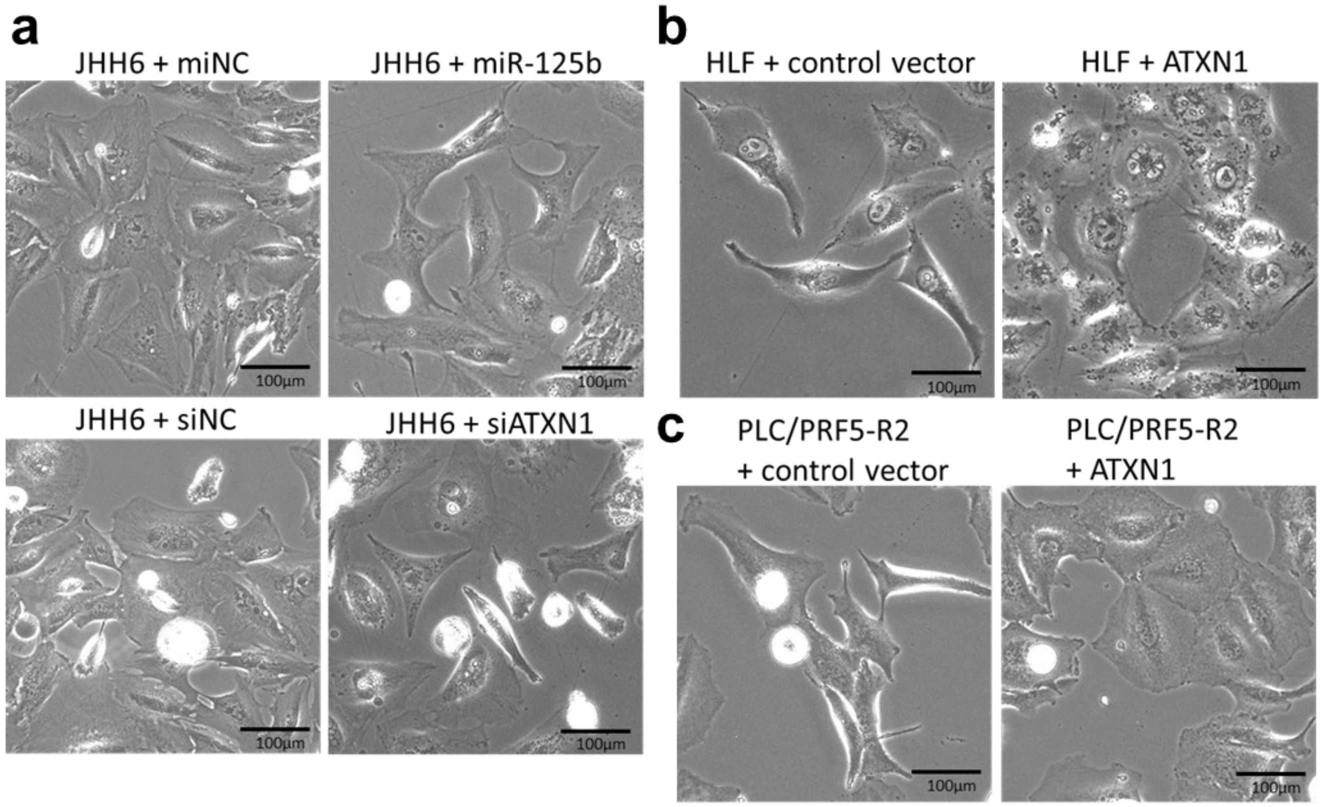

Figure S6. The phenotypical changes of HCC cell lines treated with miR-125b-5p mimic or siRNA-ATXN1, ATXN1 expression vector. (**a**) Representative images of JHH6-miNC, JHH6-miR125b, JHH6-siNC and JHH6-siATXN1 cells under observation with a stereoscopic microscope are shown. Scale bars, 100 μm; (**b**) Representative images of HLF cells treated with control vector or ataxin1 expression vector; (**c**) Representative images of PLC/PRF5-R2 cells treated with control vector or ataxin1 expression vector.

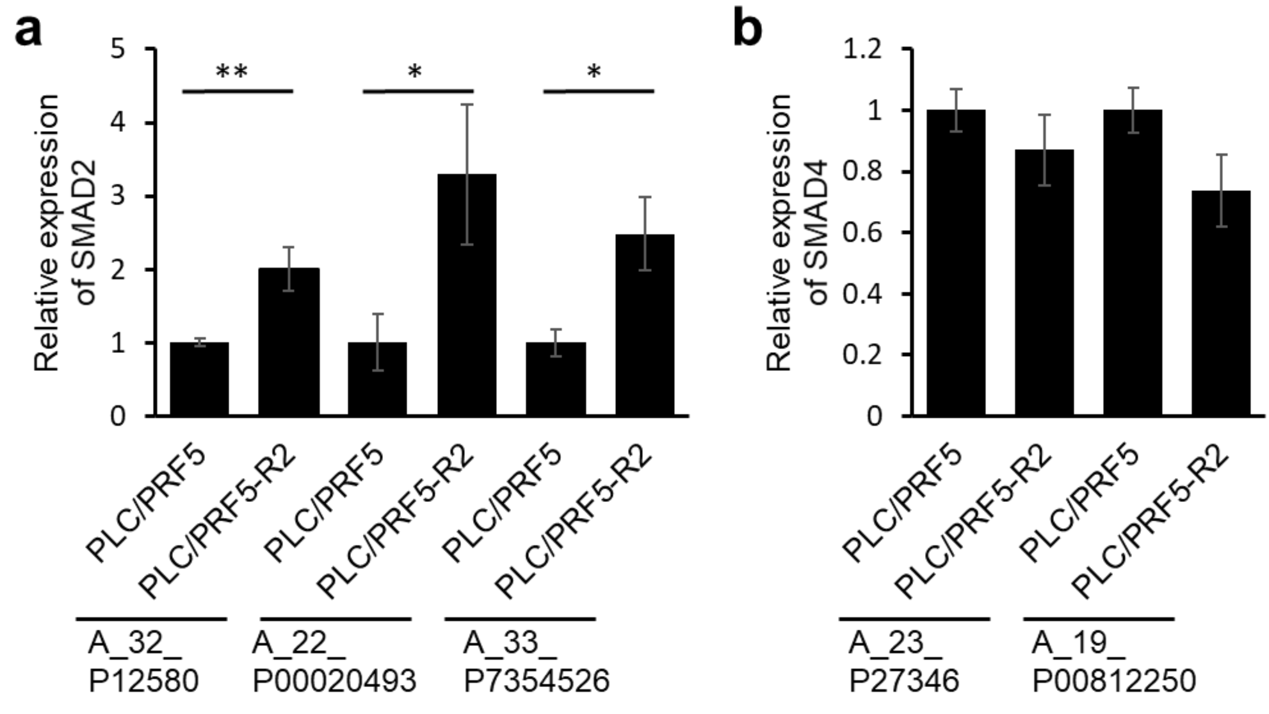

Figure S7. The SMAD2/4 expression in PLC/PRF5-R2 and PLC/PRF5 cells as revealed by microarray analysis. (**a**) The SMAD2 mRNA expression levels examined using 3 kinds of probes (A_32_P12580, A_22_P00020493, A_33_P7354526) are shown. (**b**) The SMAD4 mRNA expression levels examined using 2 kinds of probes (A_23_P27346, A_19_P00812250) are shown. *$p<0.05$, **$p<0.01$.

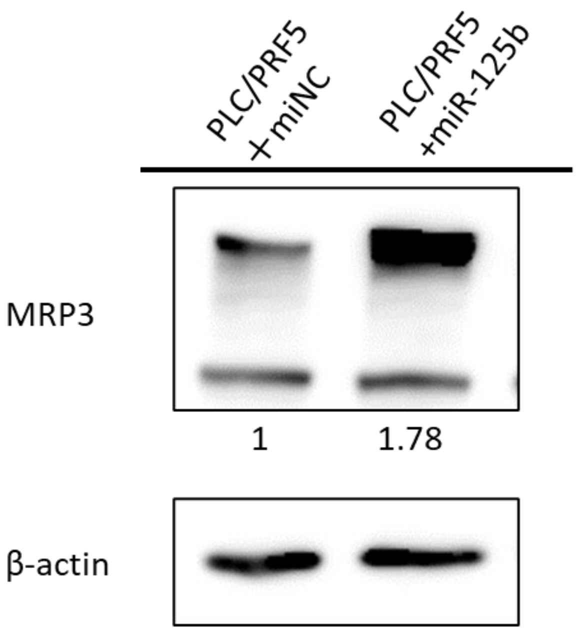

Figure S8. Expression of MRP3 in PLC/PRF5-miNC and PLC/PRF5-miR125b cells. The expression of MRP3 was examined by western blot analysis. Densitometry analysis indicates relative protein levels from 1 representative of 3 independent experiments. Numbers below the bands represent protein expression normalized to β-actin.

Fig 2c E-cad/Snail/B-actin

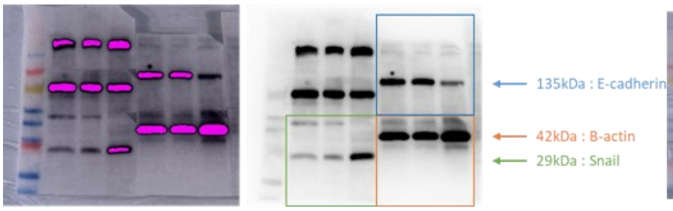

Fig 2c Vimentin/B-actin

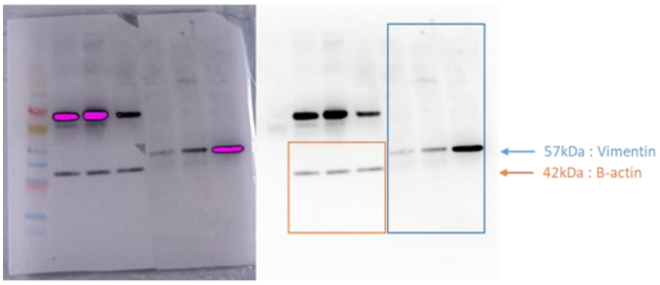

Fig3e

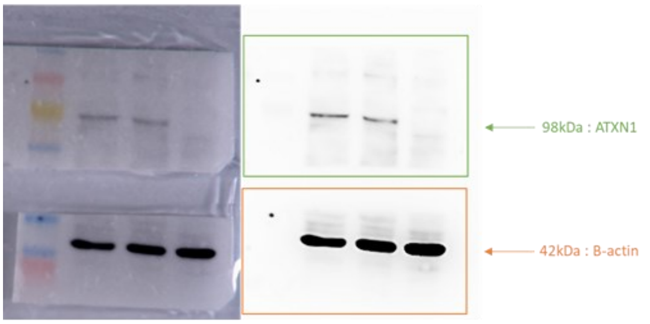

Fig 4a ATXN1/Vimentin/B-actin

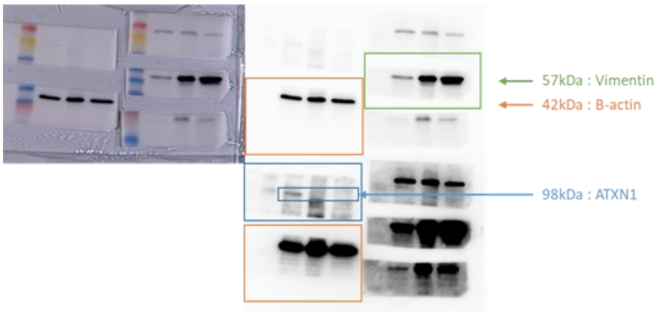

Fig 4a Snail/B-actin

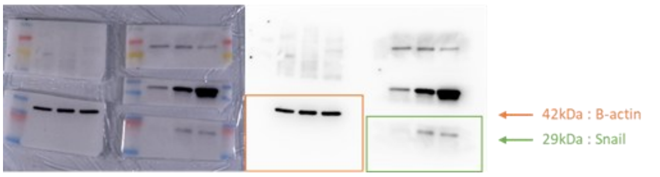

Fig 4a E-cadherin/B-actin

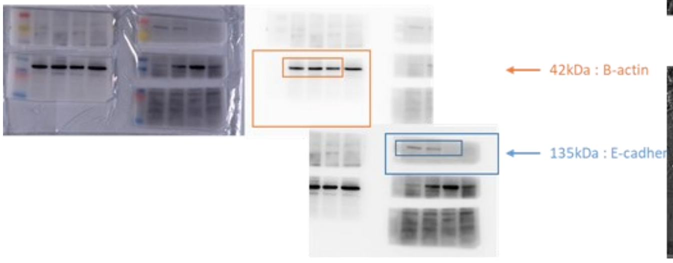

Fig 6c E-cadherin, B-actin, Snail, ATXN1

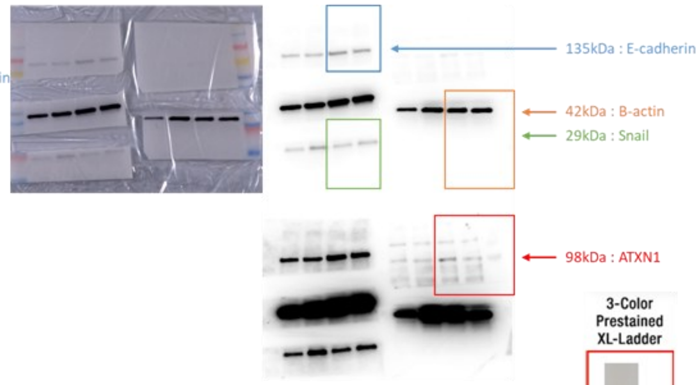

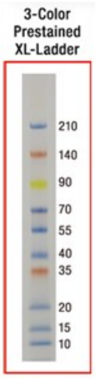

Fig 6c, d Vimentin

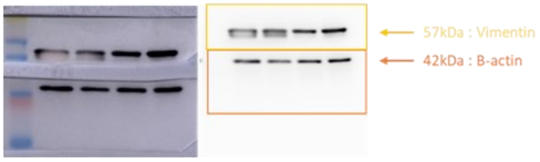

Fig 6d E-cadherin/Snail/B-actin

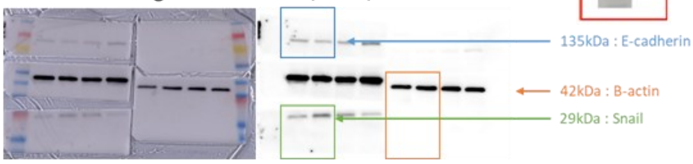

Fig 6d ATXN1/B-actin

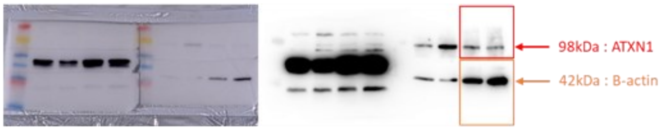

Fig 6g,h E-cadherin/Vimentin/B-actin

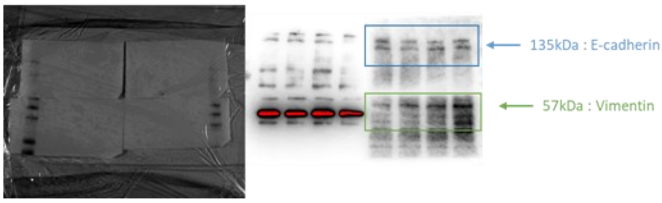

Fig 6g,h Snail/B-actin

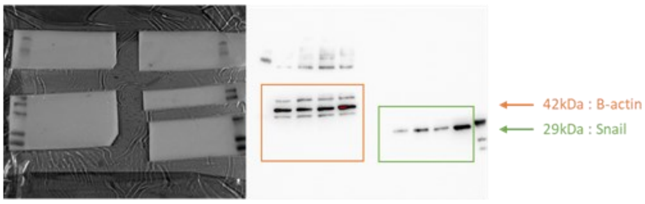

Fig 6g,h ATXN1

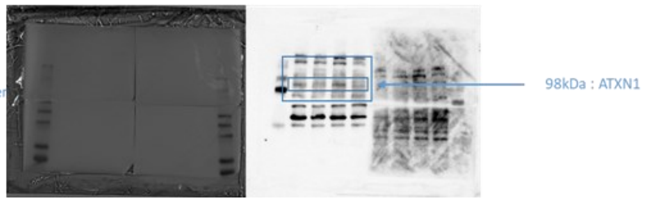

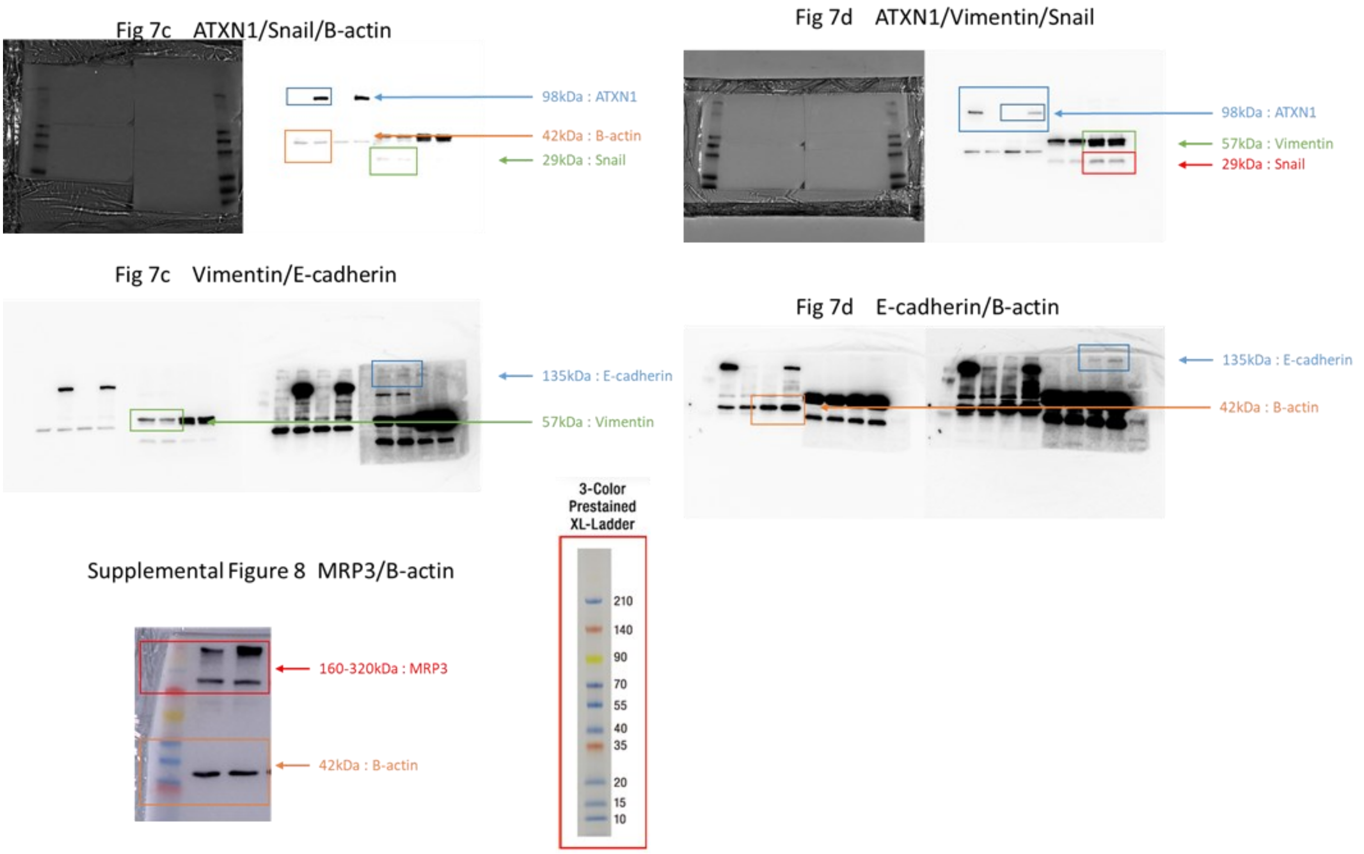

Figure S9. Uncropped western blot images.

Table S1. Taqman Assay of each miRNAs and genes

Reagents			source
TaqMan™ MicroRNA Assay	hsa-miR-100-5p	000437	Applied Biosystems
TaqMan™ MicroRNA Assay	hsa-miR-125b-5p	000449	Applied Biosystems
TaqMan™ MicroRNA Assay	hsa-miR-192-5p	000491	Applied Biosystems
TaqMan™ MicroRNA Assay	hsa-miR-193b-3p	002367	Applied Biosystems
TaqMan™ MicroRNA Assay	hsa-miR-194-5p	000493	Applied Biosystems
TaqMan™ MicroRNA Assay	hsa-miR-210-3p	000512	Applied Biosystems
TaqMan™ MicroRNA Assay	hsa-miR-215-5p	000518	Applied Biosystems
TaqMan™ MicroRNA Assay	U6	001973	Applied Biosystems
TaqMan® Gene Expression Assays	ATXN1	Hs00165656_m1	Applied Biosystems

Table S2. miRNA mimics and siRNAs

Reagents			source
mirVana® miRNA mimic	hsa-miR-100-5p	MIMAT0000098	Applied Biosystems
mirVana® miRNA mimic	hsa-miR-125b-5p	MIMAT0000423	Applied Biosystems
mirVana® miRNA mimic	hsa-miR-192-5p	MIMAT0000222	Applied Biosystems
mirVana® miRNA mimic	hsa-miR-193b-3p	MIMAT0002819	Applied Biosystems
mirVana® miRNA mimic	hsa-miR-194-5p	MIMAT0000460	Applied Biosystems
mirVana® miRNA mimic	hsa-miR-210-3p	MIMAT0000267	Applied Biosystems
mirVana® miRNA mimic	hsa-miR-215-5p	MIMAT0000272	Applied Biosystems
mirVana® miRNA mimic	Negative Control #1		Applied Biosystems
Silencer Select	ATXN1	s12488	Applied Biosystems
Halo Tag® Control Vector	Negative Control		Promega
pFN21AB7066	ATXN1	KIBB7066	Promega
MISSION® Lenti microRNA	hsa-miR-125b-5p	MIMAT0000423	Sigma-Aldrich
MISSION® Lenti microRNA	Negative Control 1		Sigma-Aldrich

Table S3. Primer sets

Primers for cloning the ATXN1 promoter region		
		primer sequences (5' - 3')
	forward	AAAAAAGCTAGCGGTGAGGGTACCTGTGCAGT
	reverse	AAAAAAAAGCTTGTAAATGGATCTGGGGTTGC

Oligonucleotides of *ATXN1* 3’UTR inserted to psiCHECK2 vector		
miR-125b-5p binding site 1		sequences (5' - 3')
Wild type	forward	TCGAGCTTGCTTAATCTTCCATATATTCTGCTCAGGG-<br>CACTTGCAATTATTAGGTTTTGTTTGC
	reverse	GGCCGCAAACAAAACCTAATAATT-<br>GCAAGTGCCCTGAGCAGAATATATGGAAGATTAA-<br>GCAAGC
Mutant 1	forward	TCGAGCTTGCTTAATCTTCCATATATTCTGA-<br>GACTTTCACTTGCAATTATTAGGTTTTGTTTGC
	reverse	GGCCGCAAACAAAACCTAATAATT-<br>GCAAGTGAAAGTCTCAGAATATATGGAAGATTAA-<br>GCAAGC
Mutant 2	forward	TCGAGCTTGCTTAATCTTCCATATATTCTGGAG-<br>TCCCCACTTGCAATTATTAGGTTTTGTTTGC
	reverse	GGCCGCAAACAAAACCTAATAATTGCAAGTGGG-<br>GACTCCAGAATATATGGAAGATTAAGCAAGC
miR-125b-5p binding site 2		sequences (5' - 3')
Wild type	forward	TCGAGTCTGCGGAACCTTTCACAC-<br>CTCTTTCTCAGGGACGGGGCAGGTGTGTGTGTGG-<br>TACACGC
	reverse	GGCCGCGTGTACCACACACACAC-<br>CTGCCCCGTCCCTGAGAAA-<br>GAGGTGTGAAAGGTTCCGCAGAC
Mutant 1	forward	TCGAGTCTGCGGAACCTTTCACACCTCTTTA-<br>GACTTTACGGGGCAGGTGTGTGTGTGGTACACGC
	reverse	GGCCGCGTGTACCACACACACACCTGCCCCG-<br>TAAAGTCTAAAGAGGTGTGAAAGGTTCCGCAGAC
Mutant 2	forward	TCGAGTCTGCGGAACCTTTCACACCTCTTTGAG-<br>TCCCACGGGGCAGGTGTGTGTGTGGTACACGC
	reverse	GGCCGCGTGTACCACACACACACCTGCCCCGTGG-<br>GACTCAAAGAGGTGTGAAAGGTTCCGCAGAC

Table S4. High expression of 6 miRNAs in PLC/PRF5-R1 compared with parent cells

Systematic name	mirbase accession No	FC (PLC vs R1)[1]
hsa-miR-100-5p	MIMAT0000098	241.1368
hsa-miR-125b-5p	MIMAT0000423	34.69279
hsa-miR-193b-3p	MIMAT0002819	2.345073
hsa-miR-210-3p	MIMAT0000267	2.263242
hsa-miR-494-3p	MIMAT0002816	2.027126
hsa-miR-7641	MIMAT0029782	2.035562

[1] Fold change in expression of miRNA between PLC/PRF5 and PLC/PRF5-R1

Table S5. High expression of 7 miRNAs in PLC/PRF5-R2 compared with parent cells

Systematic name	mirbase accession No	FC (PLC vs R2)[1]
hsa-miR-100-5p	MIMAT0000098	2085.995
hsa-miR-125b-5p	MIMAT0000423	379.0602
hsa-miR-193b-3p	MIMAT0002819	4.418106
hsa-miR-210-3p	MIMAT0000267	4.213196
hsa-let-7a-5p	MIMAT0000062	2.94759
hsa-let-7e-5p	MIMAT0000066	3.843901
hsa-let-7f-5p	MIMAT0000067	2.755421

[1] Fold change in expression of miRNA between PLC/PRF5 and PLC/PRF5-R2

Table S6. Potential targets of miR-125b-5p

Target Rank	Target Score	Gene Symbol
1	99	FREM1
2	99	PPP4R3A
3	99	SH3TC2
4	99	BMF
5	99	STARD13
6	99	ZNF704
7	99	GCNT1
8	99	ARID3B
9	98	IER3IP1
10	98	BLZF1
11	97	KLF13
12	97	CGN
13	97	NUP210
14	97	NIPAL4
15	97	IRF4
16	97	RORA
17	97	DOCK3
18	97	KCNS3
19	97	TMEM135
20	97	NBEAL2
21	97	BAK1
22	97	ACHE
23	97	KCNK10
24	96	JADE2
25	96	PI4K2B
26	96	ZNF543
27	96	CYP24A1
28	96	ZSCAN29
29	96	KCTD15
30	96	ZSWIM6
31	96	NPL
32	96	TTPA
33	95	ETS1
34	95	SLC37A2
35	95	SBNO1
36	95	PRTG
37	95	KIAA1841
38	95	ANAPC16
39	95	OSBPL9
40	95	LIPA
41	95	SSTR3
42	95	MTF1
43	95	DUS1L
44	95	VPS4B
45	95	CDH5
46	95	NECAB3
47	95	ENPEP
48	94	SLC39A9
49	94	ATXN1
50	94	SEL1L
